# Outcomes from a Driving and Community Mobility Intervention Designed for Novice Drivers with Autism from the Perspective of the Participants and Their Parents

**DOI:** 10.1007/s10803-024-06618-6

**Published:** 2024-10-19

**Authors:** Anne E. Dickerson, Lynne Murphy, Mary McIntyre

**Affiliations:** Department of Occupational Therapy, East Carolina University, Greenville, USA

**Keywords:** Driving, Community mobility, Autism, Executive function, Anxiety

## Abstract

To examine change in driving and community mobility outcomes for teens and young adults with autism as a result of participating in an occupational therapy intervention designed as a Bootcamp as perceived by the participants and their parents. Matched questionnaires were completed by novice drivers with autism as well as their parents prior to and immediately after the intervention. The intervention consisted of a 5-day (32 h) intervention using interactive driving simulators, role playing, and highly interactive learning experiences. Sixty-seven participants and their parents completed the pre and post surveys. Of these, 52 (80%) were male and 13 (20%) were female, with a mean age of 17.8 *±* 3.03 years. Wilcoxon signed rank tests was used for the Likert scale questions and paired t test for ratio level data. Results demonstrated participants perceived significant improvement in knowledge, skills and abilities related to both driving and community mobility. There were also significant differences in perception from the parents’ perspective, but not as evident as the participants. Only a few significant changes were perceived in terms of executive functioning, which support accuracy of the results. Findings also showed significantly improvement in anxiety and confidence.As driving and community mobility is critical for young adults with autism to be successful in adult roles, intervention for improving knowledge, skills, and abilities in this complex daily task is essential. This study demonstrates statistically significant outcomes of a driving and community mobility occupational therapy intervention from the perspective of the participants and their parents.

## Introduction

As young adults, the deficits associated with autism spectrum disorder affect instrumental activities of daily living (IADL), including learning to drive. In examining autistic young adults with autism perspective on independence (Cheak-Zamora et al., 2022), researchers found a key factor in the “pursuit of independence” was the ability to drive. Specifically, participants viewed driving as a way of becoming more self-determined and reducing the feeling of burdening others. Moreover, in a study comparing the relationship of employment with external factors (Zalewska et al., 2016), it was found that autistic people with a means of community mobility have five times higher odds of being employed compared to their peers who do not.

Although many people with autism obtain their driver’s license, they do so significantly later than drivers who do not have autism. In fact, one in three autistic teens attain a license compared to 83.5% of neurotypical teens (Curry et al., 2018). From the viewpoint of autistic teens, parents, and driving instructors (Cox et al., 2012; Vindin et al., 2021), barriers to learning to drive included issues with anxiety, cognitive and executive function deficits, motor coordination, motor planning, multi-tasking, social communication, motivation and/or confidence. In addition, Vindin and colleagues (2021) examined external challenges that included parents’ negative impact on the process as well as greater costs and time for autistic individuals learning to drive. The study concluded there is a clear need for a specialized model of training for novice drivers with autism. Similarly, in another review (Kersten et al., 2020), the researchers identified needs and barriers to driving and/or using public transportation. They also emphasized the importance of specialized learning strategies, practice, and support for novice drivers with autism. Not surprising, without such specialized services, integration into higher education, joining the workforce and independent living will be impacted for these young adults (Turcotte et al., 2016). Unfortunately, few programs exist (Wilson et al., 2018) and insufficient number of studies address strategies that improve the driving capabilities of autistic novice drivers (Lindsay, 2017). However, one such occupational therapy intervention, a 5-day driving and community mobility “bootcamp” for autistic teens and young adults, demonstrated significant improvements with driving capabilities, decreasing their anxiety about driving, and meeting self-identified driving and community mobility objectives in a recent study (Dickerson et al., 2024). As a program spanning seven iterations, there are multiple research questions to yet be addressed. Thus, this paper will examine the perceptions of the intervention outcomes by the autistic participants and their parents by comparing their responses prior to and after the same 5-day (approximately 32 h) intervention. The specific research questions included: *Are there significant improvement in driving and community mobility knowledge*,* skills*,* and abilities in autistic teens and young adults due to an occupational therapy intervention designed as a Bootcamp as perceived by the participants and their parents?*

## Methods

### Design

A pre-test, post-test analysis was used to compare the perceptions of change from the 5-day intervention from the perspective of the participants and their parents. All pretesting was done one to four weeks prior to the intervention with post-testing being done on the last day or within a week of the completion of the program. Performance-based outcomes were examined in a prior publication (Dickerson et al., 2024) and are not discussed in this paper.

### Participants

Inclusion criteria for participation in the intervention were that the participants had to be at least 14 years old, motivated to participate and self-disclosed a diagnosis of autism spectrum disorder (ASD). Participants were recruited from contacts in the community (e.g., autism support groups, medical providers), website information, and previous participants. The study was approved by East Carolina University and Medical Center Institutional Review Board and all participants signed a consent form. If the participant was 17 years old or younger, the parent or guardian provided consent for the participant and the participant signed an assent, according to IRB guidelines.

In North Carolina, teens who are 14.5 years old are eligible to take a school-supported driver’s education course and obtain their permit at 15 years of age after passing the state licensing agency’s written test and on road assessment.

### Instrument

Using the online questionnaire *Qualtrics*^™^ platform, pre and post questionnaires were developed to collect both the parents’ and participants’ perception of change resulting from the intervention. There were specific questions grouped in five categories with five-point Likert scale. The categories included: (1) Knowledge (e.g., understand rules and regulations, identify components of a car) with (e.g., 1 = strongly disagree to 5 = strongly agree); (2) Driving Ability Skills (e.g., use mirrors appropriately, successfully maintain lane position) with (e.g., 1 = poor to 5 = excellent); (3) Executive Functioning Skills (e.g., planning movements, multi-tasking) with (e.g., 1 = strongly disagree to 5 = strongly agree); (4) Future Abilities (e.g., be an independent driver, use public transportation safely) with (e.g., 1 = strongly disagree to 5 = strongly agree) and (5) Barriers to Driving (e.g., lack of interest, anxiety) with (e.g., 1 = not a barrier to 5 = always a barrier). Individual questions included level of anxiety for the participant and parent as well as if the Bootcamp met their expectations. Both pre and post surveys also included open-ended questions.

### Intervention

The intervention was established in 2015 and continued each year (except for 2017 and 2020) with data for this analysis collected over all the years of the intervention. Four to eight occupational therapy master’s level students assist in evaluation and implementation of the program under the leadership of the principal investigator, an occupational therapy faculty researcher. The intervention consists of planned individual and group activities designed to build or improve driving and community mobility related skills. These include knowledge-based sessions related to pre-driving skills and community mobility skills using active learning strategies (e.g., scavenger hunts, mapping games), sessions on interactive driving simulators and visual scanning boards, using stationary vehicles for orientation and demonstration of vehicle parts, an interactive visit with law enforcement officers, role playing (e.g., getting a ticket, using ride hailing, the visit to get your license), and riding a community bus. A detailed description is available elsewhere along with a description of the model of implementation fidelity (Dickerson et al., 2024).

### Data Analysis

Pre and post surveys were downloaded into databases, matching the participants and parents by an ID code. Wilcoxon signed rank tests was used for the Likert scale questions and paired t test for ratio level data. Using key words to categorize and group responses, four of the participant’s open-ended questions were counted. Data was analyzed using SPSS (Version 29, IBM) and with a significance level of 0.05.

## Results

### Participants

Each of the seven intervention groups (years) had a range of 7 to 14 participants for a total of 67 participants who finished both the pre and post surveys. Of the group, 52 (80%) were male and 13 (20%) were female, with a mean age of 17.8 *±* 3.03 years and a range from 14 to 30 years of age. The largest percent of the participants finished 12th grade (*N* = 25, 38.5%) and 11th grade (*N* = 12, 18.5%) but ranged from 8th grade to 2 years of college. It was equally divided between participants who finished driver’s education (*N* = 33, 50.8%), but the majority did not have a driver’s permit (*N* = 43, 66.2%) and only three participants had a driver’s license (4.6%).

Table 1 shows the number and percentage for pre and post survey questions about *Knowledge* about driving and community mobility and the statistical comparison from the perspective of the parent and participant. Table 2 shows the number and percentage for pre and post survey questions about in *Driving Ability* and the statistical comparison from the perspective of the parent and participant. Table 3 shows the number and percentage for pre and post survey questions about *Executive Functions skills* and the statistical comparison from the perspective of the parent and participant. Table 4 shows the number and percentage for pre and post survey questions about *Barriers for driving* and the statistical comparison from the perspective of the parent and participant. Table 5 shows the parents and participants perception of anxiety prior to and after the intervention. Table 6 summarizes participant comments for select open ended questions. Finally, of the 70 parents, 59 (84.3%) indicated the intervention exceeded or far exceeded their expectations. Of the 66 participants, 48 (72.8%) indicated the bootcamp exceeded or far exceeded their expectations with 16 (24.2%) selecting “meets expectations” and only 2 (3%) selecting it meeting short or far short of their expectations.

**Table 1:** Parents’ and participants’ perception of change in knowledge about driving and community mobility. For each statement, the parents and participants indicated their level of agreement

Perception of Change	*N*	*n* (%)						Paired *n*	*p*	
		Strongly disagree	Disagree	Neutral	Agree	Strongly Agree	Not Applicable			
**Parents: “My child is able to...”**										
Identify the components of a car used for driving (e.g., brake, gas, gearshift).								68	0.088	
Pretest	68	1 (1.5)	6 (8.8)	4 (5.9)	26 (38.2)	31 (45.6)	0			
Posttest	68	1 (1.5)	2 (2.9)	3 (4.4)	25 (36.8)	37 (54.4)	0			
Understand traffic rules and regulations.								67	**< 0.001**	
Pretest	67	2 (3.0)	9 (13.4)	13 (19.4)	29 (43.3)	12 (17.9)	2 (3.0)			
Posttest	69	1 (1.4)	0	2 (2.9)	34 (49.3)	32 (46.4)	0			
Be a safe and adequate driver.								67	**< 0.001**	
Pretest	67	5 (7.5)	16 (23.9)	21 (31.3)	23 (34.3)	2 (3.0)	0			
Posttest	67	1 (1.5)	6 (9.0)	16 (23.9)	29 (43.3)	14 (20.9)	1 (1.5)			
Obtain a driver’s license								63	0.104	
Pretest	64	1 (1.6)	7 (10.9)	14 (21.9)	34 (53.1)	8 (12.5)	0			
Posttest	63	1 (1.6)	1 (1.6)	22 (34.9)	19 (30.2)	19 (30.2)	1 (1.6)			
Read a map.								66	**< 0.001**	
Pretest	66	6 (9.1)	22 (33.3)	6 (9.1)	20 (30.3)	11 (16.7)	1 (1.5)			
Posttest	66	1 (1.5)	5 (7.6)	15 (22.7)	31 (47.0)	14 (21.2)	0			
Use GPS/smartphone to find my way.								66	**0.024**	
Pretest	68	3 (4.4)	6 (8.8)	10 (14.7)	26 (38.2)	21 (30.9)	2 (2.9)			
Posttest	66	1 (1.5)	3 (4.5)	3 (4.5)	28 (42.4)	31 (47.0)	0			
Use public transportation.								62	**< 0.001**	
Pretest	62	9 (14.5)	20 (32.3)	16 (25.8)	10 (16.1)	6 (9.7)	1 (1.6)			
Posttest	66	0	3 (4.5)	19 (28.8)	26 (39.4)	17 (25.8)	1 (1.5)			
Safely navigate within the neighborhood (walking)								59	0.078	
Pretest	59	1 (1.7)	6 (10.2)	7 (11.9)	16 (27.1)	29 (49.2)	0			
Posttest	59	1 (1.7)	0	6 (10.2)	20 (33.9)	32 (54.2)	0			
Use a taxi service								61	**< 0.001**	
Pre	61	9 (14.8)	20 (32.8)	15 (24.6)	9 (14.8)	7 (11.5)	1 (1.6)			
Post	61	1 (1.6)	3 (4.9)	19 (31.1)	17 (27.9)	20 (32.8)	1 (1.6)			
**Participants: “I am able to...”**										
Identify the components of a car used for driving (e.g., brake, gas, gearshift).								65	**0.008**	
Pretest	65	0	3 (4.6)	6 (9.2)	30 (46.2)	26 (40.0)	0			
Posttest	65	1 (1.5)	2 (3.1)	1 (1.5)	20 (30.8)	41 (63.1)	0			
Understand traffic rules and regulations.								65	**0.001**	
Pretest	65	0	4 (6.2)	9 (13.8)	34 (52.3)	17 (26.2)	1 (1.5)			
Posttest	65	0	0	2 (3.1)	31 (47.7)	32 (49.2)	0			
Be a safe and adequate driver.								65	**0.010**	
Pretest	65	1 (1.5)	4 (6.2)	11 (16.9)	31 (47.7)	18 (27.7)	0			
Posttest	65	0	2 (3.1)	7 (10.8)	30 (46.2)	26 (40.0)	0			
Obtain a driver’s license								62	**0.010**	
Pretest	63	3 (4.8)	4 (6.3)	16 (25.4)	18 (28.6)	21 (33.3)	1 (1.6)			
Posttest	62	0	2 (3.2)	10 (16.1)	27 (43.5)	21 (33.9)	2 (3.2)			
Read a map.								64	**< 0.001**	
Pretest	64	5 (7.8)	12 (18.8)	15 (23.4)	17 (26.6)	13 (20.3)	2 (3.1)			
Posttest	65	2 (3.1)	2 (3.1)	10 (15.4)	30 (46.2)	20 (30.8)	1 (1.5)			
Use GPS/smartphone to find my way.								64	**0.021**	
Pretest	64	0	0	8 (12.5)	24 (37.5)	32 (50)	0			
Posttest	65	0	0	2 (3.1)	22 (33.8)	41 (63.1)	0			
Use public transportation.								59	**< 0.001**	
Pretest	59	2 (3.4)	6 (9.2)	14 (23.7)	29 (49.2)	8 (13.6)	0			
Posttest	65	2 (3.1)	2 (3.1)	4 (6.2)	24 (36.9)	33 (50.8)	0			
Safely navigate within the neighborhood (walking).								45	**0.005**	
Pretest	54	0	2 (3.7)	7 (13.0)	25 (46.3)	20 (37.0)	0			
Posttest	55	0	1 (1.8)	4 (7.3)	15 (27.3)	35 (63.6)	0			
Use a taxi service.								59	**0.002**	
Pretest	59	6 (10.2)	8 (13.6)	19 (32.2)	21 (35.6)	4 (6.8)	1 (1.7)			
Posttest	65	3 (4.6)	7 (10.8)	13 (20.0)	23 (35.4)	18 (27.7)	1 (1.5)			

**Table 2:** Parents’ and participants’ perception of change in child’s driving ability skills. For each statement, the parents and participants indicated their perceptions of ability

Perception of Change	*N*	*n* (%)					Paired *n*	*p*
		Poor	Fair	Good	Very Good	Excellent		
**Parents: “I believe my child’s ability to do each of the following is...”**								
Successfully maintain lane position							53	**< 0.001**
Pretest	53	5 (9.4)	17 (32.1)	25 (47.2)	6 (11.3)	0		
Posttest	60	0	6 (10.0)	26 (43.3)	23 (38.3)	5 (8.3)		
Successfully control the speed of the car							52	**< 0.001**
Pretest	52	3 (5.8)	19 (36.5)	20 (38.5)	6 (11.5)	4 (7.7)		
Posttest	60	0	10 (16.7)	21 (35.0)	21 (35.0)	8 (13.3)		
Successfully brake in response to a stimuli							53	**0.001**
Pretest	53	7 (13.2)	25 (47.2)	12 (22.6)	7 (13.2)	2 (3.8)		
Posttest	60	0	20 (33.3)	18 (30.0)	16 (26.7)	6 (10.0)		
Use the mirrors appropriately							53	**< 0.001**
Pretest	53	9 (17.0)	21 (39.6)	14 (26.4)	9 (17.0)	0		
Posttest	60	1 (1.7)	13 (21.7)	23 (38.3)	16 (26.7)	7 (11.7)		
Be aware of traffic situations and respond appropriately							53	**< 0.001**
Pretest	53	16 (30.2)	22 (41.5)	12 (22.6)	3 (5.7)	0		
Posttest	60	1 (1.7)	21 (3.50)	22 (36.7)	13 (21.7)	3 (5.0)		
Make turns appropriately at traffic lights							53	**< 0.001**
Pretest	53	6 (11.3)	18 (34.0)	22 (41.5)	6 (11.3)	1 (1.9)		
Posttest	60	0	15 (25.0)	23 (38.3)	15 (25.0)	7 (11.7)		
Make a right turn at an intersection without an indicator, safely							53	**< 0.001**
Pretest	53	7 (13.2)	16 (30.2)	23 (43.4)	5 (9.4)	2 (3.8)		
Posttest	60	1 (1.7)	11 (18.3)	29 (48.3)	13 (21.7)	6 (10.0)		
Make a left turn at an intersection without an indicator, safely							53	**< 0.001**
Pretest	53	15 (28.3)	16 (30.2)	18 (34.0)	4 (7.5)	0		
Posttest	60	3 (5.0)	20 (33.3)	24 (40.0)	9 (15.0)	4 (6.7)		
Successfully park in a parking lot							53	**< 0.001**
Pretest	53	13 (24.5)	19 (35.8)	17 (32.1)	3 (5.7)	1 (1.9)		
Posttest	60	3 (5.0)	12 (20.0)	28 (46.7)	15 (25.0)	2 (3.3)		
Yield to other cars and pedestrians							53	**< 0.001**
Pretest	53	4 (7.5)	19 (35.8)	19 (35.8)	7 (13.2)	4 (7.5)		
Posttest	60	0	10 (16.7)	26 (43.3)	17 (28.3)	7 (11.7)		
Use turn signals consistently							52	**< 0.001**
Pretest	52	3 (5.8)	14 (26.9)	18 (34.6)	14 (26.9)	3 (5.8)		
Posttest	60	0	5 (8.3)	22 (36.7)	20 (33.3)	13 (21.7)		
Back up Safely							53	**< 0.001**
Pretest	53	14 (26.4)	20 (37.7)	11 (20.8)	6 (11.3)	2 (3.8)		
Posttest	59	1 (1.7)	21 (35.6)	23 (39.0)	10 (16.9)	4 (6.8)		
Appropriately maintain distance between vehicles							53	**< 0.001**
Pretest	53	4 (7.5)	22 (41.5)	15 (28.3)	10 (18.9)	2 (3.8)		
Posttest	59	0	7 (11.9)	27 (45.8)	16 (27.1)	9 (15.3)		
Obey traffic regulations							53	**< 0.001**
Pretest	53	2 (3.8)	9 (17.0)	25 (47.2)	12 (22.6)	5 (9.4)		
Posttest	60	0	4 (6.7)	21 (35.0)	23 (38.3)	12 (20.0)		
**Participants: “I believe my ability to do each of the following is...”**								
Successfully maintain lane position							59	**< 0.001**
Pretest	59	3 (5.1)	17 (28.8)	20 (33.9)	16 (27.1)	3 (5.1)		
Posttest	65	1 (1.5)	7 (10.8)	20 (30.8)	27 (41.5)	10 (15.4)		
Successfully control the speed of the car							58	**< 0.001**
Pretest	58	7 (12.1)	15 (25.9)	21 (36.2)	13 (22.4)	2 (3.4)		
Posttest	65	0	10 (15.4)	27 (41.5)	18 (27.7)	10 (15.4)		
Successfully brake in response to a stimuli							59	**< 0.001**
Pretest	59	5 (8.5)	12 (20.3)	26 (44.1)	13 (22.0)	3 (5.1)		
Posttest	65	0	5 (7.7)	19 (29.2)	26 (40.0)	15 (23.1)		
Use the mirrors appropriately							59	**< 0.001**
Pretest	59	2 (3.4)	9 (15.3)	31 (52.5)	15 (25.4)	2 (3.4)		
Posttest	65	1 (1.5)	9 (13.8)	18 (27.7)	22 (33.8)	15 (23.1)		
Be aware of traffic situations and respond appropriately							56	**< 0.001**
Pretest	58	5 (8.6)	11 (19.0)	28 (48.3)	12 (20.7)	2 (3.4)		
Posttest	56	0	9 (13.8)	19 (29.2)	26 (40.0)	11 (16.9)		
Make turns appropriately at traffic lights							59	**0.002**
Pretest	59	6 (10.2)	11 (18.6)	27 (41.5)	12 (20.3)	3 (4.6)		
Posttest	65	2 (3.1)	7 (10.8)	23 (35.4)	19 (29.2)	14 (21.5)		
Make a right turn at an intersection without an indicator, safely							59	**< 0.001**
Pretest	59	9 (15.3)	23 (39.0)	14 (23.7)	11 (18.6)	2 (3.4)		
Posttest	65	2 (3.1)	10 (15.4)	21 (32.3)	21 (32.3)	11 (16.9)		
Make a left turn at an intersection without an indicator, safely							58	**< 0.001**
Pretest	58	11 (19.0)	24 (41.4)	13 (22.4)	8 (13.8)	2 (3.4)		
Posttest	65	3 (4.6)	14 (21.5)	25 (38.5)	12 (18.5)	11 (16.9)		
Successfully park in a parking lot							58	0.072
Pretest	58	7 (12.1)	18 (31.0)	20 (34.5)	10 (17.2)	3 (5.2)		
Posttest	65	6 (9.2)	18 (27.7)	21 (32.3)	11 (16.9)	9 (13.8)		
Yield to other cars and pedestrians							58	**0.001**
Pretest	59	2 (3.4)	11 (18.6)	25 (42.4)	15 (25.4)	6 (10.2)		
Posttest	58	1 (1.7)	3 (5.2)	20 (34.5)	19 (32.8)	15 (25.9)		
Use turn signals consistently							57	**0.005**
Pretest	59	5 (8.5)	7 (11.9)	23 (39.0)	13 (22.0)	11 (18.6)		
Posttest	57	0	4 (7.0)	17 (29.8)	18 (31.6)	18 (31.6)		
Back up Safely							58	**0.003**
Pretest	59	12 (20.3)	18 (30.5)	21 (35.6)	5 (8.5)	3 (5.1)		
Posttest	58	4 (6.9)	14 (24.1)	17 (29.3)	17 (29.3)	6 (10.3)		
Appropriately maintain distance between vehicles							58	**0.016**
Pretest	59	3 (5.1)	19 (32.2)	18 (30.5)	12 (20.3)	7 (11.9)		
Posttest	58	2 (3.4)	6 (10.3)	16 (27.6)	24 (41.4)	10 (17.2)		
Obey traffic regulations							48	**0.002**
Pretest	49	4 (8.2)	7 (14.3)	12 (24.5)	17 (34.7)	9 (18.4)		
Posttest	48	0	1 (2.1)	11 (22.9)	20 (41.7)	16 (33.3)		

**Table 3:** Parents’ and participants’ perception of change in executive functioning skills. For each statement, the parents and participants indicated their level of agreement

Perception of Change	*N*	*n* (%)					Paired *n*	*p*
		Strongly disagree	Disagree	Neutral	Agree	Strongly Agree		
**Parents: “I believe my child’s has difficulty with...”**								
Planning Movements							68	0.467
Pretest	68	1 (1.5)	22 (32.4)	19 (27.9)	19 (27.9)	7 (10.3)		
Posttest	69	4 (5.8)	12 (17.4)	19 (27.5)	31 (44.9)	3 (4.3)		
Multitasking							69	0.686
Pretest	69	2 (2.9)	8 (11.6)	11 (15.9)	35 (50.7)	13 (18.8)		
Posttest	71	2 (2.8)	6 (8.5)	18 (25.4)	33 (46.5)	12 (16.9)		
Focusing							70	0.782
Pretest	70	2 (2.9)	11 (15.7)	16 (22.9)	32 (45.7)	9 (12.9)		
Posttest	71	4 (5.6)	9 (12.7)	12 (16.9)	38 (53.5)	8 (11.3)		
Attention							69	0.661
Pretest	69	2 (2.9)	13 (18.8)	14 (20.3)	35 (50.7)	5 (7.2)		
Posttest	71	4 (5.6)	11 (15.5)	14 (19.7)	33 (46.5)	9 (12.7)		
Following 1–2 command verbal directions							60	0.080
Pretest	61	3 (4.9)	27 (44.3)	10 (16.4)	20 (32.8)	1 (1.6)		
Posttest	60	5 (8.3)	18 (30.0)	7 (11.7)	25 (41.7)	5 (8.3)		
Following multi-step verbal directions							70	0.313
Pretest	70	2 (2.9)	15 (21.4)	14 (20.0)	27 (38.6)	12 (17.1)		
Posttest	71	3 (4.2)	15 (21.1)	17 (23.9)	28 (39.4)	8 (11.3)		
Tolerating changes in routine							69	0.590
Pretest	69	5 (7.2)	16 (23.2)	9 (13.0)	33 (47.8)	6 (8.7)		
Posttest	71	3 (4.2)	18 (25.4)	19 (26.8)	25 (35.2)	6 (8.4)		
Tolerating when others make mistakes							70	0.581
Pretest	70	4 (5.7)	24 (34.3)	13 (18.6)	26 (37.1)	3 (4.3)		
Posttest	71	4 (5.6)	26 (36.6)	16 (22.5)	22 (31.0)	3 (4.2)		
Adapting to changes in my environment							70	0.906
Pretest	70	4 (5.7)	16 (22.9)	16 (22.9)	29 (41.4)	5 (7.1)		
Posttest	71	5 (7.0)	16 (22.5)	15 (21.1)	31 (43.7)	4 (5.6)		
Controlling impulses							70	0.363
Pretest	70	4 (5.7)	24 (34.3)	21 (30.0)	20 (28.6)	1 (1.4)		
Posttest	70	6 (8.6)	21 (30.0)	18 (25.7)	18 (25.7)	7 (10.0)		
Time Management							70	0.740
Pretest	70	3 (4.3)	14 (20.0)	15 (21.4)	25 (35.7)	13 (18.6)		
Posttest	70	2 (2.9)	16 (22.9)	18 (18.6)	32 (45.7)	7 (10.0)		
Problem Solving							70	0.610
Pretest	70	1 (1.4)	16 (22.9)	13 (18.6)	35 (50.0)	5 (7.1)		
Posttest	70	4 (5.7)	17 (24.3)	13 (18.6)	28 (40.0)	8 (11.4)		
Anticipating consequences							69	0.084
Pretest	69	2 (2.9)	24 (34.8)	14 (20.3)	26 (37.7)	3 (4.3)		
Posttest	71	3 (4.2)	12 (16.9)	22 (31.0)	26 (36.6)	8 (11.3)		
Generalizing information or learning							64	**0.046**
Pretest	64	4 (6.3)	18 (28.1)	16 (25.0)	24 (37.5)	2 (3.1)		
Posttest	70	3 (4.3)	17 (24.3)	11 (15.7)	32 (45.7)	7 (10.0)		
**Participants: “I have difficulty with...”**								
Planning Movements							55	**0.041**
Pretest	55	6 (10.9)	16 (29.1)	13 (23.6)	16 (29.1)	4 (7.3)		
Posttest	56	11 (19.6)	16 (28.6)	14 (25.0)	13 (23.2)	2 (3.6)		
Multitasking							63	0.681
Pretest	63	5 (7.9)	13 (20.6)	19 (30.2)	17 (27.0)	9 (14.3)		
Posttest	63	6 (9.5)	16 (25.4)	16 (25.4)	14 (22.2)	11 (17.5)		
Focusing							63	0.372
Pretest	65	4 (6.2)	14 (21.5)	18 (27.7)	20 (30.8)	9 (13.8)		
Posttest	63	7 (11.1)	16 (25.4)	16 (25.4)	15 (23.8)	9 (14.3)		
Attention							62	**0.014**
Pretest	62	4 (6.5)	15 (24.2)	17 (27.4)	15 (24.2)	11 (17.7)		
Posttest	65	10 (15.4)	21 (32.3)	11 (16.9)	15 (23.1)	8 (12.3)		
Following 1–2 command verbal directions							50	0.994
Pretest	50	6 (12.0)	21 (42.0)	11 (22.0)	9 (18.0)	3 (6.0)		
Posttest	53	10 (18.9)	17 (32.1)	13 (24.5)	9 (17.0)	4 (7.5)		
Following multi-step verbal directions							62	1.000
Pretest	62	5 (8.1)	17 (27.4)	11 (17.7)	19 (30.6)	10 (16.1)		
Posttest	64	5 (7.8)	15 (23.4)	14 (21.9)	23 (35.9)	7 (10.9)		
Tolerating changes in routine							62	0.151
Pretest	62	1 (1.6)	24 (38.7)	12 (19.4)	20 (32.3)	5 (8.1)		
Posttest	63	11 (17.5)	20 (31.7)	10 (15.9)	17 (27.0)	5 (7.9)		
Tolerating when others make mistakes							63	0.293
Pretest	63	8 (12.7)	23 (36.5)	11 (17.5)	12 (19.0)	9 (14.3)		
Posttest	64	10 (15.6)	24 (27.5)	13 (20.3)	12 (18.8)	5 (7.8		
Adapting to changes in my environment							63	0.909
Pretest	65	8 (12.3)	24 (36.9)	12 (18.5)	18 (27.7)	3 (4.6)		
Posttest	63	11 (17.5)	19 (30.2)	14 (22.2)	15 (23.8)	4 (6.3)		
Controlling impulses							62	**0.034**
Pretest	62	5 (8.1)	21 (33.9)	10 (16.1)	21 (33.9)	5 (8.1)		
Posttest	64	18 (28.1)	16 (25.0)	12 (18.8)	10 (15.6)	8 (12.5)		
Time Management							62	0.524
Pretest	62	7 (11.3)	13 (21.0)	14 (22.6)	19 (30.6)	9 (14.5)		
Posttest	63	10 (15.9)	14 (22.2)	8 (12.7)	23 (36.5)	8 (12.7)		
Problem Solving							62	0.199
Pretest	62	9 (14.5)	22 (35.5)	8 (12.9)	17 (27.4)	6 (9.7)		
Posttest	64	13 (20.3)	23 (35.9)	11 (17.2)	10 (15.6)	7 (10.9)		
Anticipating consequences							65	0.673
Pretest	65	13 (20.0)	23 (35.4)	9 (13.8)	14 (21.5)	6 (9.2)		
Posttest	64	10 (15.6)	26 (40.6)	9 (14.1)	11 (17.2)	8 (12.5)		
Generalizing information or learning							61	0.300
Pretest	61	13 (21.3)	25 (41.0)	9 (14.8)	9 (14.8)	5 (8.2)		
Posttest	65	20 (30.8)	21 (32.3)	9 (13.8)	11 (16.9)	4 (6.2)		

**Table 4:** Parents’ and participants’ perception of change in barriers to child’s ability to be a driver. For each statement below, the parent identified which are the barriers for their child

Perception of Change	*N*	*n* (%)				Paired *n*	*p*
		Not a barrier	Sometimes a barrier	Frequently a barrier	Always a Barrier		
**Parents: “Barriers to my child’s ability to drive include...”**							
Anxiety tied to operating a car						62	0.086
Pretest	67	15 (22.4)	23 (34.3)	21 (31.3)	8 (11.9)		
Posttest	62	16 (25.8)	27 (43.5)	13 (21.0)	6 (9.7)		
Fear of crashing						59	0.701
Pretest	67	19 (28.4)	25 (37.3)	14 (20.9)	9 (13.4)		
Posttest	59	16 (27.1)	26 (44.1)	10 (16.9)	7 (11.9)		
Previous crash/collision						61	0.863
Pretest	66	56 (84.8)	3 (4.5)	3 (4.5)	4 (6.1)		
Posttest	61	51 (83.6)	6 (9.8)	2 (3.3)	2 (3.3)		
Lack of comprehension of road rules and regulations						62	**0.002**
Pretest	67	26 (38.8)	29 (43.3)	10 (14.9)	2 (3.0)		
Posttest	62	31 (50.0)	27 (43.5)	3 (4.8)	1 (1.6)		
Parental limitation (not giving permission)						62	0.585
Pretest	67	36 (53.7)	17 (25.4)	10 (14.9)	4 (6.0)		
Posttest	62	31 (50.0)	22 (35.5)	7 (11.3)	2 (3.2)		
Lack of driving training opportunities						62	0.140
Pretest	66	25 (37.9)	15 (22.7)	14 (21.2)	12 (18.2)		
Posttest	62	19 (30.6)	27 (43.5)	13 (21.0)	3 (4.8)		
Lack of time for driving training						62	0.919
Pretest	66	33 (50.0)	17 (25.8)	6 (9.1)	10 (15.2)		
Posttest	62	28 (45.2)	16 (25.8)	14 (22.6)	4 (6.5)		
Lack of experience driving						63	0.053
Pretest	66	12 (18.2)	18 (27.3)	11 (16.7)	25 (27.9)		
Posttest	63	14 (22.2)	14 (22.2)	26 (41.3)	8 (12.7)		
Lack of interest						62	0.828
Pretest	67	36 (53.7)	18 (26.9)	10 (14.9)	3 (4.5)		
Posttest	62	32 (51.6)	21 (33.9)	7 (11.3)	2 (3.2)		
Lack of focus						61	0.449
Pretest	67	19 (28.4)	27 (40.3)	18 (26.9)	3 (4.5)		
Posttest	61	17 (27.9)	31 (50.8)	12 (19.7)	1 (1.6)		
Lack of financial resources						62	0.175
Pretest	67	50 (74.6)	11 (16.4)	4 (6.0)	2 (3.0)		
Posttest	62	51 (82.3)	8 (12.9)	2 (3.2)	1 (1.6)		
Don’t feel the need to drive						62	0.156
Pretest	66	38 (57.6)	19 (28.8)	7 (10.6)	2 (3.0)		
Posttest	62	31 (50.0)	20 (32.3)	8 (12.9)	3 (4.8)		
Can’t pass drivers license exam						61	0.578
Pretest	67	42 (62.7)	10 (14.9)	10 (14.9)	5 (7.5)		
Posttest	61	38 (62.3)	13 (21.3)	7 (11.5)	3 (4.9)		
**Participants: “Barriers to my ability to drive include...”**							
Anxiety tied to operating a car						66	**0.003**
Pretest	66	21 (31.8)	21 (31.8)	12 (18.2)	12 (18.2)		
Posttest	67	28 (41.8)	28 (41.8)	6 (9.0)	5 (7.5)		
Fear of crashing						66	**0.002**
Pretest	66	12 (18.2)	20 (30.3)	16 (24.2)	18 (27.3)		
Posttest	67	15 (22.4)	33 (49.3)	8 (11.9)	11 (16.4)		
Previous crash/collision						65	0.272
Pretest	65	48 (73.8)	7 (10.8)	3 (4.6)	6 (9.2)		
Posttest	66	51 (77.3)	7 (10.6)	6 (9.1)	2 (3.0)		
Lack of comprehension of road rules and regulations						65	**0.005**
Pretest	66	34 (51.5)	20 (30.3)	7 (10.6)	5 (7.6)		
Posttest	65	43 (66.2)	18 (27.7)	3 (4.6)	1 (1.5)		
Parental limitation (not giving permission)						66	0.397
Pretest	66	37 (56.1)	13 (19.7)	10 (15.2)	6 (9.1)		
Posttest	67	39 (58.2)	15 (22.4)	5 (7.5)	8 (11.9)		
Lack of driving training opportunities						65	0.776
Pretest	65	31 (47.7)	20 (30.8)	10 (15.4)	4 (6.2)		
Posttest	66	30 (45.5)	18 (27.3)	13 (19.7)	5 (7.6)		
Lack of time for driving training						65	0.061
Pretest	65	27 (41.5)	18 (27.7)	16 (24.6)	4 (6.2)		
Posttest	66	31 (47.0)	23 (34.8)	10 (15.2)	2 (3.0)		
Lack of experience driving						65	0.327
Pretest	65	20 (30.8)	17 (26.2)	16 (24.6)	12 (18.5)		
Posttest	67	19 (28.4)	25 (37.3)	11 (16.4)	12 (17.9)		
Lack of interest						65	0.874
Pretest	65	46 (70.8)	13 (20.0)	6 (9.2)	0		
Posttest	66	46 (69.7)	16 (24.2)	2 (3.0)	2 (3.0)		
Lack of focus						65	0.234
Pretest	65	29 (44.6)	24 (36.9)	9 (13.8)	3 (4.6)		
Posttest	66	31 (47.0)	28 (42.4)	5 (7.6)	2 (3.0)		
Lack of financial resources						65	0.703
Pretest	65	40 (61.5)	12 (18.5)	10 (15.4)	3 (4.6)		
Posttest	67	39 (58.2)	19 (28.4)	6 (9.0)	3 (4.5)		
Don’t feel the need to drive						65	**0.041**
Pretest	65	44 (67.7)	15 (23.1)	3 (4.6)	3 (4.6)		
Posttest	66	34 (51.5)	24 (36.4)	4 (6.1)	4 (6.1)		
Can’t pass drivers license exam						65	**0.002**
Pretest	65	41 (63.1)	9 (13.8)	7 (10.8)	8 (12.3)		
Posttest	65	51 (78.5)	9 (13.8)	4 (6.2)	1 (1.5)		

**Table 5:** Parents and participants perception of anxiety at pre and posttest and comparison of change

	*N*		*n* (%)							Paired *n*	*p*
			Not at all anxious	A little Anxious		Somewhat Anxious	Very Anxious	Extremely Anxious			
**Parents**: Which of the following best describes your level of anxiety when you think about your son/daughter becoming a driver.											
Pretest	56	1 (1.8)			6 (10.7)	16 (28.6)	26 (32.5)		7 (8.8)	50	0.167
Posttest	55	0			10 (18.2)	20 (36.4)	18 (41.9)		3 (7.0)		
**Parents**: Which of the following best describes your son or daughter’s level of anxiety with becoming a driver.											
Pretest	43	2 (4.7)			7 (16.3)	13 (30.2)	18 (11.5)		4 (7.7)	39	**0.002**
Posttest	55	4 (7.3)			17 (30.9)	24 (43.6)	10 (18.2)		10 (18.2)		
**Participants**: Which of the following best describes your level of anxiety when you think about becoming a driver											
Pretest	52	6 (11.5)			19 (36.5)	19 (36.5)	5 (9.6)		3 (5.8)	52	**0.019**
Posttest	52	9 (17.3)			26 (50.0)	10 (19.2)	7 (13.5)		0		

**Table 6:** Participant comments to opened ended questions: pre and Post Bootcamp (inclusive of 2016, 2018, 2019, 2021, 2022, 2023)

Questions	*N*	
Pre: What expectations do you have of the Bootcamp?	45	Learn to be a good, safe, independent driver; improve my driving*
	4	No expectations
	3	Learn as much as I can
	3	More confidence to pass the test to drive
	2	Have fun
	2	Handle when I get upset; anxiety
Post: What did you enjoy the most about bootcamp?	27	Driving simulator
	16	Social environment, new friends
	12	All the activities, games, learning**
	10	Vision Coach
	8	Bus trip
	7	My skills improving
	3	Law enforcement visit
Post: What was the best outcome for you as a result of participating in the bootcamp?	16	Better driver, learning to drive, seeing improvement
	13	More knowledge, awareness of rules, pedestrians, learning signs.
	12	More confidence/less anxiety/overcome fears
	6	Making new friends
	4	Read/use a map
Post: As a result of the bootcamp, what skills do you think improved?	19	Improved passing, turning, merging, maneuvers
	11	Identifying traffic signs or signals/ rules
	8	Better attention/focus
	6	Maps and navigation
	6	Road hazards
	5	Controlling speed and brakes
	4	Community mobility
	4	Confidence

*control car like speed limit, lane position, be aware of surrounding and quicker response to stimuli, advice for tough traffic situations, ability to focus, how to be behind the wheel, develop new and old skills, how to react to others, steering, how to turn, safety, traffic rules

**different ways of public transportation, traffic signs and rules, how to respond to hazards, parts of the car, the courses, reaction time, road signs, scenarios and what to do, tips and expectations on the road, re-orient to driving regulations

### Discussion

The objective of this study was to examine the efficacy of an intervention to improve driving and community mobility skills, knowledge, and abilities of autistic novice drivers from the perceptive of the participants and their parents. Clearly, most of the parents and participants responded that the intervention was positive and met or exceeded expectations suggesting this occupational therapy intervention was perceived as effective. The outcomes from most of the questionnaires also are positive. For example, both parents and participants surveys indicated they perceived significant improvement in driving abilities after the intervention. These included maintaining lane position, using the brake and accelerator appropriately, making appropriate turns, using mirrors, obeying traffic regulations, and responding to other traffic. While the participants could observe their skills improving, only about 36% of the parents had the opportunity to observe actual changes as their child had a driving permit. Nevertheless, many parents acknowledged the improvement in knowledge and skills as in this comment from a parent: *“He now believes he has the basic skills to drive and wants to have more behind the wheel and real driving practice. This was not the case prior to attending bootcamp”* and *“He has asked to practice driving regularly. We began yesterday! He has not been willing to drive in the last 6 months.”* The one driving maneuver participants did not show a significant change was “successfully park in a parking lot” which was not surprising as our driving simulators do not have the capacity to execute that maneuver and actually supports the fidelity of the analysis.

In terms of knowledge about driving and community mobility, it was expected that there would be perceived changes in all areas of knowledge based on the amount of education provided. This was true for participants, although not as evident for the parents. While there was some shifting to more agreement, apparently most of the parents apparently assumed their children were able to “identify the components of a car for driving” and “safely navigate within the neighborhood” prior to the intervention. Interestingly, the participants felt they learned more than what parents perceived; potentially due to the learning process used in the intervention. For example, to learn components of the car, the participants were in vehicles and asked to demonstrate competency using the various components of the vehicle, reinforcing the knowledge through active learning (Yannier et al., 2021). Learning to *safely* navigate in the neighborhood was reinforced through using maps and/or smartphones, as well as safety precautions when using ride sharing and using the bus through the active learning of performing the methods of navigating. Most significant active learning likely occurred using the interactive driving simulators especially after reinforcement of rules of the road in games or activities designed to learn traffic strategies (e.g., using miniature cars on traffic mats).

A hallmark for people with autism are deficits in executive functioning (Wallace et al., 2016). While often gifted with high intelligence, autistic people often have difficulty with multi-tasking, planning, tolerating changes in route, tolerating others’ mistakes, problem solving, anticipating consequences, and generalizing information or learning. As expected, parents did not perceive any changes in the executive skills listed, except for *generalizing learning*. In this case, generalizing the ability to understand rules of the road in more than just one place has been identified as an issue in driving (Cox et al., 2012). Even though just a week, parents may have seen changes in the generalization of learning as exemplified in a comment by one parent in the post survey question about change: “*He’s also been paying more attention to road signs and seems to pay more attention to the routes we take when driving. Previously*,* he had no interest in these things*.”

Interestingly, the participants perceived change in three other characteristics often identified in as issues in autism: *attention*,* impulse control* and *planning movement.* Both attention and impulse control were targeted through the driving simulator and Vision Coach^™^ intervention strategies. For example, one strategy for Vision Coach was to hit the red buttons with letters but not numbers (Hatfield et al. 2018) and attention was critical in identifying potential hazards with both the driving simulator and hazard activities. In terms of motor planning, a graded step-by-step process was used to build the skills for steering and using the pedals, with success required at each step facilitating the achievement of motor coordination. Thus, the participants did achieve improved motor planning. However, regardless of these exceptions, most of the characteristics of executive function were not perceived as changed, offering strong support that participants’ and parents’ perceptions were truthful and accurate, that is, the intervention was not seen as a solution for all issues experienced by autistic individuals.

Multiple studies have identified barriers to driving for autistic teens (Almberg et al., 2017; Kersten et al., 2020; Ross et al., 2018). In our study, most parents did not perceive many of the common (as frequently or always a barrier) barriers found in previous studies. The barrier, “lack of experience driving,” is understandable and in this case, although not reaching significant, there was a major shift respondents to lower numbers in the category of “always a barrier.” The barrier that did show a significant change was “lack of comprehension of road rules and regulations” (from more to less of a barrier). This barrier also showed the same significant change by the participants. Both these results were expected as rules of the road was a major emphasis of the intervention. However, in contrast to their parents, participants shifted in their perception of barriers in several key areas, including: anxiety tied to operating a vehicle, fear of crashing, don’t feel the need to drive, and can’t pass the driver’s license exam. Changes in these four barriers are significant supporting the intervention is effective not only in improving driving knowledge, skills, and abilities, but also decreasing anxiety and increasing confidence.

Anxiety about driving, in particular, has been found as a key issue in most studies (Chee et al., 2015; Kersten et al., 2020; Lindsay, 2017; Vindin et al., 2021), including our assessments for both participants and parents. Thus, we added an individual question about anxiety. Matching the barrier outcome, the participants described their anxiety significantly decreasing while the parents remained the same. What is interesting here is that parents were asked to describe the level of anxiety of their son or daughter. In this case, the change in anxiety was also significant, but in the opposite direction – shifting to be more anxious. There is not a clear explanation for this result. It is possible the participants talked about being anxious during the bootcamp, but ultimately felt more prepared to succeed, thus less anxious when doing the post-assessment. Another possibility for this result may be more of a reflection of the parents’ anxiety upon the participants’ increased positivity about wanting to drive and being able to pass the licensing exam. This is an important avenue for further investigation.

When asked in an open-ended question, 66% of the participants responded with comments about wanting to improve their driving or learning to be a good, safe or independent driver. When asked what they enjoyed most, 40% named the driving simulator and 24% identified making new friends and/or the positive social environment. Others mentioned the games and learning activities, using Vision Coach^™^, and “seeing my skills improve.” When asked what the best outcome from the bootcamp was, 24% specifically mentioned seeing their improvement, being a better driver, or learning to drive. Other outcomes included more confidence, less anxiety, increased driving knowledge (e.g., awareness of rules, learning signs, pedestrian rules) and making new friends. Finally, 28% of the participants indicated that improvement in performing maneuvers (e.g., passing, turning, merging) as the skills that most improved. Other skills mentioned included identifying traffic signs, signals, and rules; better attention; learning maps and navigation; and hazard detection.

### Limitations

This study has the limitation of being based on perceptions rather than evidence based on performance. However, the outcomes in this study collaborate the previously published changes in performance (Dickerson et al., 2024). Additionally, the limited number of significant changes in perception of executive functions, which would not be expected, support the significant changes in perception. Another limitation is that questions can be misinterpreted, or outcomes need more information or description. Future work with qualitative interviews may capture more of this kind of information. Finally, a major limitation is that there is no actual on-road experience, which is not realistically possible due to most participants not having their permit. However, several parents have contacted the program to share positive stories about their son or daughter well after they finished the program. In fact, two different parents shared a similar story about their son’s first crash. One story is shared here:

*Yesterday*,* Billy*^1^*was in his first accident*,* which was not his fault. I am writing to let you know how extremely proud we are of how he handled the entire situation … and I fully believe it was because of your camp. Not only was he rear-ended and pushed into another car*,* but the driver of the at-fault vehicle fled the scene. He called me first*,* then used the SOS button in his car to call police. He was perfectly calm*,* gave descriptions of the cars involved and even provided a description of the man who left the scene. Once the police arrived*,* he gave his information and he repeated what happen. His car was drivable*,* and he hopped right back in to drive home. This happened in a construction zone on… a main road at 4:30 pm*,* so traffic was crazy. Thank you …for offering this bootcamp!*

## Conclusion

Unfortunately, there is a significant decrease in healthcare service accessibility and utilization for autistic young adults once they exit high school and/or age out of insurance coverage from parents (Kennedy-Hendricks et al., 2018; Turcotte et al., 2016). Thus, since driving underlies the ability to seek, gain and retain employment, autistic teens and young adults may have no insurance or are underinsured since they are no longer on their parents’ plan, limiting their options for health equity as well as work. This study contributes to demonstrating that appropriate occupational therapy intervention for autistic novice drivers can be successful and improve the outcomes for independent driving and/or community mobility. As driving and community mobility is an instrumental activity of daily living within the scope of occupational therapy, this should be a call to action for practitioners to provide such services.

## Footnotes

1 Not his real name.
